# Resibufogenin suppresses colorectal cancer growth and metastasis through RIP3-mediated necroptosis

**DOI:** 10.1186/s12967-018-1580-x

**Published:** 2018-07-20

**Authors:** Qinrui Han, Ye Ma, Hao Wang, Yu Dai, Chunhui Chen, Yawei Liu, Linlin Jing, Xuegang Sun

**Affiliations:** 1 Laboratory of Molecular Medicine, School of Traditional Chinese Medicine, Southern Medical University, Guangzhou, 510515 Guangdong China; 2 The Laboratory for Neurosurgery, Nanfang Hospital, Southern Medical University, Guangzhou, China; 3 TCM Integrated Hospital of Southern Medical University, Guangzhou, China

**Keywords:** Resibufogenin, Necroptosis, Metastasis, Receptor-interacting protein kinase 3, Colorectal cancer

## Abstract

**Background:**

Necroptotic susceptibility is probably an intrinsic weakness of cancer. Here, we report that resibufogenin, a member of bufadienolide family, suppresses the growth and metastasis of colorectal cancer (CRC) through induction of necroptosis in vivo.

**Methods:**

SW480 cells with stably expressing enhanced green fluorescence protein were xenografted to BALB/c-nu mice to observe the growth of tumors. Liver metastasis was observed by injection of MC38 cells beneath the splenic capsule of mice. Protein expression was determined by immunohistochemistry, immunofluorescence and western blot.

**Results:**

Consolidated in vitro results indicate that resibufogenin has anti-proliferative activity on CRC cells. PI staining and transmission electron microscope imaging suggest that the cell death induced by resibufogenin are mainly through necrosis, which is further confirmed by the ineffectiveness of z-VAD, a pan-caspase general inhibitor. In particular, resibufogenin induced necrosis is substantially abrogated in receptor-interacting protein kinase 3 (RIPK3) knockout mouse embryo fibroblasts. The RIP3-dependent necrosis has been classified as necroptosis. Resibufogenin triggeres necroptosis through upregulating RIP3 and phosphorylating mixed lineage kinase domain-like protein at Ser358. Resibufogenin also activates the expression of PYGL, GLUD1 and GLUL in a RIP3-dependent manner. Resibufogenin exerts cytotoxic effect by inducing reactive oxygen species accumulation which can be neutralized by *N*-acetylcysteine. Remarkably, resibufogenin significantly suppresses liver-metastasis from spleen implantation. The anti-neoplastic effect of this compound can be abrogated by RIP3 knockdown.

**Conclusion:**

Resibufogenin suppresses growth and metastasis of CRC through RIP3-mediated necroptosis.

**Electronic supplementary material:**

The online version of this article (10.1186/s12967-018-1580-x) contains supplementary material, which is available to authorized users.

## Background

Despite dramatic reductions in incidence and mortality for several decades, colorectal cancer (CRC) remains the third most commonly diagnosed cancer among both men and women in the United States [1]. Chemoresistance caused relapse and metastasis are the major challenges of CRC management in improving clinical outcomes [2]. The 5-year survival rate after surgical resection of colorectal metastases increases from 25 to 55%, but cancer relapse is observed in most patients [3, 4]. So, more research is needed to advance treatments for CRC with high risk of recurrence and low response rates to current therapies.

To become malignant, the colorectal epithelial cells must inactivate the apoptotic pathway to form adenomas and subsequently transform into carcinoma [5]. As a consequence, the cell’s susceptibility to apoptosis is severely compromised and other forms of death become more important for cell killing and tumour response to DNA-damaging agents. Cell death researches in recent years expand our understanding that necrosis can occur in a highly regulated and genetically controlled manner. Among them, receptor-interacting protein kinase 1 (RIP1)- and RIP3-mixed lineage kinase domain-like (MLKL)-mediated necroptosis is the most understood form of the regulated necrosis [6].

Cells evolve an elaborate programme that sense extracellular and intracellular stress signals leading to assembly of a death-inducing platform. Once the pro-apoptotic molecules failed to stimulate the apoptosome, a default death programme was triggered alternatively to induce the assembly of ripoptosome that leads to necroptosis, which relies on the kinase activity of the key enzymes RIP1 and RIP3 [7]. Previous reports have stated that some compounds such as shikonin induced a necroptotic cell death in multidrug resistant cancer cell lines to circumvent the drug resistance [8], suggesting necroptotic susceptibility is an intrinsic weakness of cancer. Thus, screening necroptosis-inducers might be a novel strategy to target the weak point of cancer [9].

Resibufogenin is a major bioactive compound that belongs to bufadienolide family. It is isolated from toad venom, which is widely used to treat malignant diseases in traditional Chinese medicine for hundreds of years. Resibufogenin is also an active ingredient of “cinobufacini injection” which is a clinical administration for advanced tumors in China [10]. It has been reported that resibufogenin increases the ratio of apoptotic cells in human hepatoma HepG2 cells [11]. A major metabolite of resibufogenin, marinobufagenin significantly induced apoptosis in A549 and H1299 cells by facilitating apoptosome assembly and caspase activation [12]. However, our results showed that the cell deaths caused by resibufogenin are mainly attributed to necroptosis. Apoptosis and necrosis are considered as different cell death entities. Nevertheless, mounting evidence suggests a common biochemical network between them termed “apoptosis-necrosis continuum” [13]. These studies enable us to discuss a rational hypothesis that resibufogenin might cause a succession of necrosis or apoptosis depending on the intensity of the insults or cellular reactivity.

Our in vivo and in vitro tests showed that resibufogenin suppresses CRC growth and metastasis by triggering RIP3-dependent necroptosis. Inducing necroptosis can be a dormant killer to be harnessed to achieve the ultimate goal of killing tumour cells [9].

## Methods

### Resibufogenin solution

Resibufogenin is a tovena lactone compound extracted from toad. The molecular formula is C_24_H_32_O_4_ with a molecular weight of 384.50 g/mol. Resibufogenin is a fat-soluble monomer, we used corn oil to dissolved or 5% DMSO plus normal saline on the oscillator to apply maximum amplitude overnight to form a drug suspension. Resibufogenin were purchased from Herbest (baoji, china), HPLC > 98%.

### Cell culture

Human CRC cell lines (SW480, HCT-116) and RIP3^+/+^ and RIP3^−/−^ (MEF) were obtained from American Type Culture Collection (ATCC; Rockville, MD, USA). SW480 and HCT-116 were incubated in RPMI-1640 medium (Invitrogen, Carlsbad, CA, USA) and RIP3^+/+^and RIP3^−/−^ were incubated in Dulbecco’s modified eagle medium (DMEM; Invitrogen). All cell lines were supplemented with 10% (v/v) fetal bovine serum (FBS; Invitrogen) and 1% (v/v) penicillin–streptomycin (Invitrogen) at 37 °C in a humidified atmosphere with 5% CO_2_.

### Xenograft CRC model

All animal research procedures were conformed to the guidelines for The Care and Use of Laboratory Animals published by the National Institutes of Health and were approved by The Laboratory Animals Care and Use Committee of Southern Medical University. SW480-eGFP (1 × 10^7^) cells were injected subcutaneously to the groin of nude mice. Ten days later, the tumors were reached to the volume of ~ 50 mm^3^ which randomly divided into groups. Animals were kept in a sterile environment. Their body weights and tumor volumes were measured every 5 days throughout the treatment period. The mice were euthanized at the end of the experiments. Tumor xenografts were weighed and photographed.

### Liver metastasis model

C57BL6/j mice (6 weeks old) were purchased from the Guangdong Experimental Animal Center (Guangdong, China). Animal study protocols were approved by The Animal Care and Experiment Committee of Southern Medical University. The liver metastasis model was created by intrasplenic injection of eGFP-MC38 cells. Briefly, the cells were slowly injected into the spleen with an insulin syringe and the blood was compressed after 3 min of hemostasis. After 2 weeks of intraperitoneal injection of resibufogenin, the fluorescence images was taken to observe the metastatic foci in liver. Tumor were weighed and photographed.

### Lentiviral preparation, viral infection, and stable cell generation

The GV248-shRNA plasmids encoding shRNAs with sequences targeting human RIP3 were purchased from the GenePharma Facility (Suzhoui, China). The shRNA-RIP3 sequence contained 5′-ACTCTCGTAATGATGTCAT-3′. The shRNA 5′-TTCTCCGAACGTGTCACGT-3′ was incorporated as a control. Cells were infected with the collected viruses over 24 h in the presence of polybrene, followed by selection in medium containing puromycin (0.5 mg/ml) for 7–9 days.

### Cell proliferation assays

Cell proliferation was measured by MTT (3-(4,5-dimethyl-2-thiazolyl)-2,5-diphenyl-2-*H*-tetrazolium bromide), according to the manufacturer’s protocol. Briefly, cancer cells (4 × 10^3^) were plated onto each well of a 96-well flat-bottomed plate and grown in RPMI medium containing 10% FBS. After 24 h, cells were treated with different concentrations of resibufogenin (0.1, 1, 2.5, 5, 10 μM) and the cells were incubated for an additional 24–48 h. The MTT (Sigma, USA, 2.5 mg/ml, 10 μl) solution was added to each well and incubated for a further 4 h. When the medium was discarded, 100 μl dimethyl sulfoxide (DMSO) was added to dissolve the formazan dye. The absorbance was read at 490 nm using multiscan spectrum.

### Cell invasion analysis

Cells from the serum-free medium (1 × 10^5^ cells/100 μl) were added to the top chamber of each 8-mm–pore transwell chamber (Corning Star; Cambridge, MA, USA). The bottom chamber was prepared using 20% FBS as a chemoattractant. Cells were allowed to migrate through the porous membrane for 48 h at 37 °C. The cells that stuck to the lower surface of the membrane were treated with a fixation/staining solution (0.1% crystal violet, 1% formalin, and 20% ethanol) for visualization. The cells were counted under a microscope in five randomly selected fields (original magnification, 200×). At least four chambers from three different experiments were analyzed.

### Cell migration analysis (wound scratch assay)

HCT116 cells were plated onto six-well culture plates in RPMI-1640 medium containing 2% FBS (2 × 10^6^ cells/well). After 24 h, the cell monolayer was scraped with a sterile 20 μl micropipette tip to create a wound, washed with PBS and photographed using Nikon inverted microscope, Thereafter, the cells were treated with resibufogenin (5 μM). After 36 h treatment period, the plates were photographed using the camera system.

### Flow cytometry

Two CRC cell lines (SW480 and HCT-116) were seeded in 6-well plates at a concentration of 1 × 10^5^ cells per well. After the cells adhered to the wall, 2 mL of resibufogenin was added to each well. A blank control group was set, then the plates were incubated for 24 or 48 h. Thereafter, the plates were analyzed by flow cytometry using a Beckman Analytical Flow Cytometer in accordance with the Flow Kit Specification (BD). A quadrant graph consisting of four quadrants was obtained and the number of cells per quadrant was the proportion of the total number of cells examined. The second quadrant represents the early apoptotic cells and the third and fourth quadrants represent the late apoptotic and necrotic cells respectively.

### LDH assay

Cell damage was determined by the release of lactate dehydrogenase (LDH) into the cell culture medium. The release of LDH was quantified using the LDH Cytotoxicity Assay Kit (Beyotime Biotechnology, CHINA) according to the manufacturer’s instructions.

### Real-time quantitative polymerase chain reaction (qRT-PCR) analysis

Total RNA was isolated from HCT116 and SW620 cells using Trizol reagent (ET101, TransGen Biotech, CHINA), cDNA was synthesized (RR037A, TAKARA BIO, JAPAN) and amplified with a PCR kit (218073, QIAGEN, Germany). qRT-PCR was repeated 3 times. GAPDH was used as the reference control. The primers used for qRT-PCR are summarized as follows: RIP3, Sense: 5-GACCTCAAGCCCTCCAATGTTC-3 and Antisense: 5-AAGTAAGCTAGGGTGCCCCCA-3; GAPDH, Sense: 5-ACCACAGTCCATGCCATCAC-3 and Antisense: 5-TCACCACCCTGTTGCTGTA-3.

### Western blot analysis

Protein expression was assessed by immunoblot analysis of cell lysates (30–60 μg) in RIPA buffer in the presence of rabbit antibodies to RIP3 (1:1000; Cell Signaling Technology), E-cadherin, fibronectin (FN), β-actin (1:500; Santa Cruz Biotech, CA, USA), GLUD1, PYGL, GLUL (1:500; Proteintech, Danvers, MA, USA), Cyt-c, and apoptosis-inducing factor (AIF) (1:500; Abcam, Cambridge, UK). The specific protein bands were visualized using an enhanced ECL system (Bio-Rad).

### Immunofluorescence

Cells were stained with MitoTracker and incubated with antibody at 4 °C overnight, then incubated with Cy3-labeled goat anti-rabbit or anti-mouse IgG antibody (1:500) in darkness for 60 min at room temperature. The cells were then counterstained with 4′,6-diamidino-2-phenylindole (DAPI) and examined under a confocal microscope (Nikon, Japan) with excitation and emission wavelengths of 550 and 570 nm, respectively, and a 100 × 1.40 NA oil immersion objective. For mitochondrial staining, 100 nM MitoTracker Green (Molecular Probes) was added to cultures 30 min before fixation.

### ROS detection

Cells were washed three times with PBS and stained with 20 μM DCFH-DA for 30 min at 37 °C and 5% CO_2_ in an incubator. The cells were trypsinized, collected by centrifugation, washed again using PBS, and re-suspended in 1 mL PBS. ROS generation was measured using flow cytometry (Beckman Analytical Flow Cytometer).

### Immunohistochemistry (IHC) staining

Tumor tissue was fixed with 4% PFA, paraffin embedded, cut into 5 μm samples. Immunohistochemical staining was conducted according to the manufacturer’s protocol. Briefly, endogenous peroxidase was blocked in a peroxidase blocking solution (0.03% hydrogen peroxide containing sodium acid) for 5 min. Tissue sections were washed gently with phosphate buffer saline (PBS, pH 7.2) and subsequently incubated with RIP3 (1:200; Abcam, Cambridge, UK), PYGL (1:50; Abcam, Cambridge, UK) and GLUL (1:200; Abcam, Cambridge, UK) and GLUD1 (1:100; Abcam, Cambridge, UK) antibodies at − 4 °C, overnight. The second day, the slides were incubated with secondary antibodies (Pre-diluted; Zhongshan Golden Bridge, Beijing, China), slides were counterstained with haematoxylin before mounting.

### Frozen sections

Tumor cryosections (5 μm-thick) were fixed for 20 min in freshly cold acetone. Sections were washed in phosphate buffered saline with 0.3% Trixton-X100 for 15 min, and then goat serum blocked for 1 h. Sections were incubated overnight at 4 °C with antibodies above. The second day, the sections were incubated for 1 h at room temperature with a goat anti-rabbit Cy2-conjugated secondary antibody (1:200, Thermo Fish, USA), After several washes in PBS the sections were mounted in Vectashield (Vector Laboratories) containing 4′,6-diamidino-2-phenyl indole (DAPI). Stained sections were imaged on confocal laser scanning microscopy.

### Statistical analyses

The results were expressed as the mean ± SEM from three independent experiments. The *P*-values were two-tailed and calculated using one-way ANOVA. Statistical significance was specified as *P *< 0.05.

## Results

### Resibufogenin suppresses the growth of heterotopic colorectal carcinoma in vivo

**Fig. 1:**
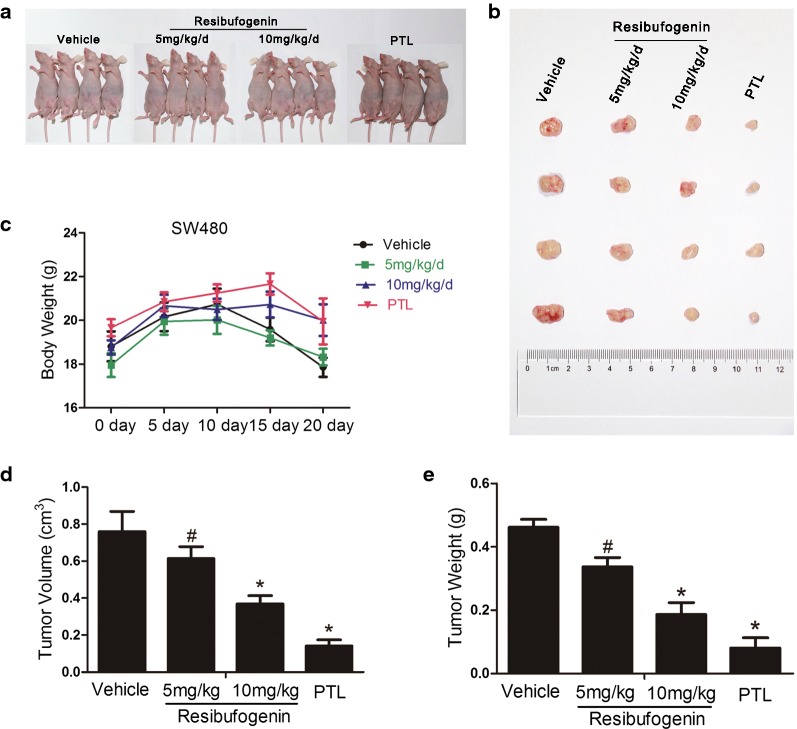
Resibufogenin suppresses the growth of heterotopic colorectal carcinoma in vivo. **a** The picture of BALB/c-nu mice with tumor. **b**, **d**, **e** Tumor size and tumor weight of the heterotropic colorectal cancer. **c** Effects of Resibufogenin and parthenolide on body weight of mice. The weight of vehicle group reduce more significantly than that of resibufogenin and PTL-treated group. Data represent mean ± SEM, n = 6. ^**#**^*P* < 0.05,*P < 0.01 as determined by one-way ANOVA followed by Tukey’s multiple comparison test

To characterize the antineoplastic role of resibufogenin, heterotropic CRC tumors derived from SW480 cells with stably expressing enhanced green fluorescence protein were xenografted to groin of BALB/c-nu mice (Fig. 1a). Parthenolide, which can induce cell necrosis through reactive oxygen species (ROS) generation, was used as a positive control in the in vivo test [9, 14, 15]. After administration (i.p.) of resibufogenin at 5 and 10 mg/kg/day in mice for 21 days, the weight of tumor in situ were significantly reduced by 27 and 41%, respectively (Fig. 1b, c). Vernier caliper measurement showed that resibufogenin decreased the tumor volume in a dose-dependent manner (Fig. 1d). The weight loss caused by tumor growth was significantly ameliorated by high dose of resibufogenin and parthenolide, respectively (Fig. 1e). These results provide strong evidence that resibufogenin suppresses tumor growth of CRC and ameliorates the weight loss in tumor-bearing mice.

### Resibufogenin induces necrosis in CRC cells

**Fig. 2:**
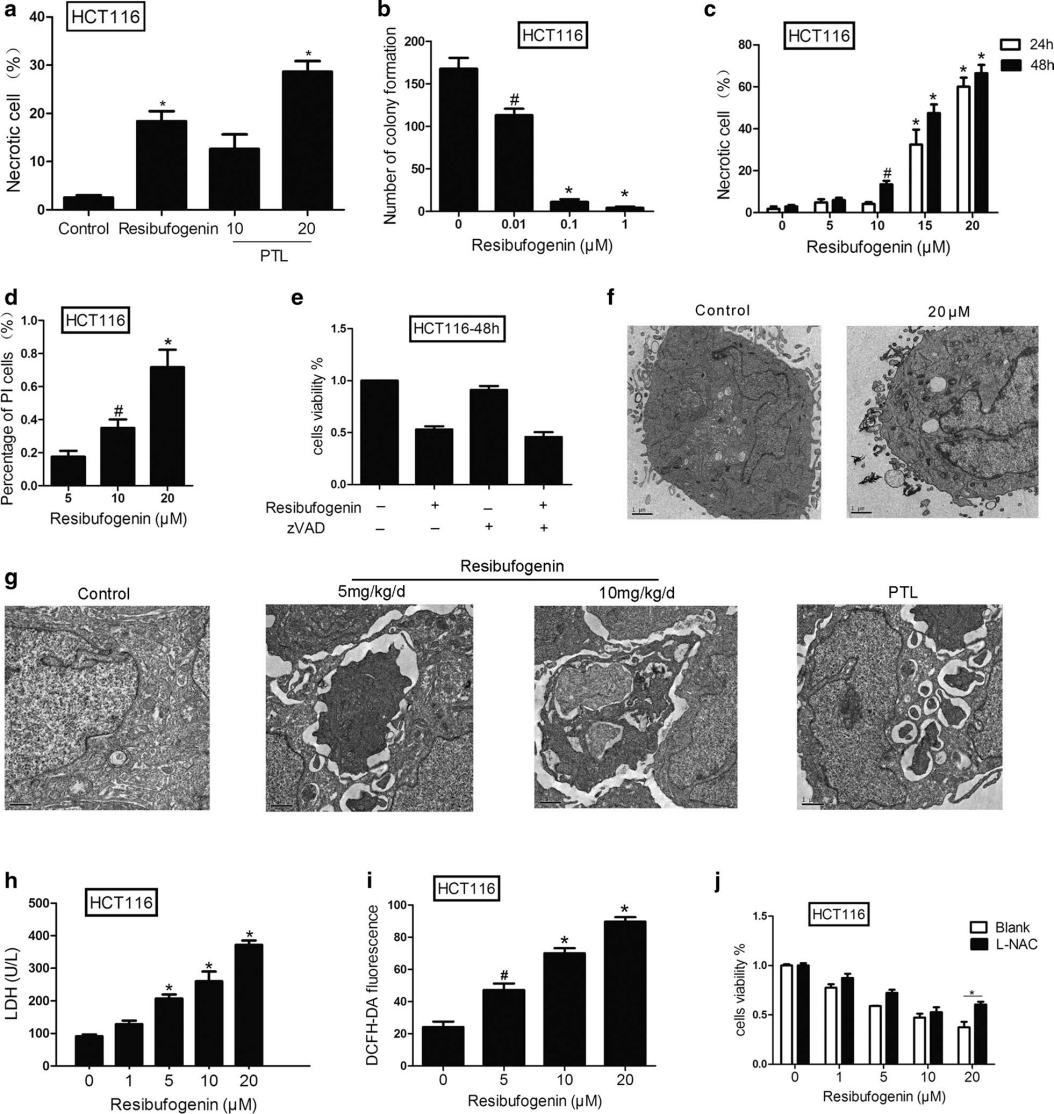
Resibufogenin induces necrosis in CRC cells. **a** Necrosis of colon cancer cells treated with PTL detected by flow cytometry at 24 h (n = 3 per group). See also Additional file 1 and Additional file 2: Figure S5. **b** Clone formation inhibition by resibufogenin from dose 0 to 1 μM in HCT116 cells. The panels represent the number of colonies as indicated. See also Additional file 1 and Additional file 2: Figure S2. **c** Flow cytometric analysis of resibufogenin treated cells with PE-annexin V/7-amino-actinomycin D staining (n = 3). Data derived from three separate experiments are presented as the mean ± SEM. See also Additional file 1 and Additional file 2: Figure S3. **d** Immunofluorescence analysis of PI-positive cells treated with resibufogenin. Cells were stained with Hoechst33342/PI (5 μg/ml) and subjected to analysis of necrosis population (n = 3). See also Additional file 1 and Additional file 2: Figure S4. **e** Cells were treated with the combination of resibufogenin and Z-VAD-fmk (20 μM). Cell viability was assayed were quantitatively analyzed (n = 6 per group). See also Additional file 1 and Additional file 2: Figure S5. **f**, **g** Morphological changes of resibufogenin-treated cells and tissues as observed by TEM. ×10,000 for all. Scale bar = 1 μm. **h** The resibufogenin-induced LDH leakage was increased in a dose-dependent manner (n = 3). **i** Effects of resibufogenin on the ROS generation of HCT116 cells (n = 3). See also Additional file 1 and Additional file 2: Figure S6. **j** The effect of resibufogenin on cell viability of colon cancer cells treated with L-NAC (n = 6). Data presented are showed as mean ± SEM from three independent experiments. *P < 0.01 indicate significant difference

Resibufogenin decreased the cell viability dose-dependently in HCT116 cells. It also lowered the cell viability mildly in IEC-6, a rat small intestinal crypt-like cell line (Additional file 1 and Additional file 2: Figure S2). The clonogenic cell survival assay showed that resibufogenin significantly reduced the number of cell clones as compared to the control group, provided further evidence that resibufogenin is an effective antineoplastic agent through inhibiting cell proliferation (Fig. 2b and Additional file 2: Figure S3) [16].

To elucidate its antineoplastic mechanism in vitro, the cell death subroutine induced by resibufogenin was identified. Annexin V/7-amino-actinomycin D double staining showed that most of the cell death induced by resibufogenin in SW480 and HCT116 cells can be classified as necrosis (Fig. 2c, d and Additional file 2: Figure S4, S6). The number of necrotic cells was significantly elevated in a dose- and time-dependent manner. Additionally, the number of necrotic cells was elevated in a dose-dependent manner treated with PTL (Fig. 2a and Additional file 2: Figure S5). Furthermore, the cell death can not be reversed by z-VAD-fmk, a pan-caspase inhibitor (Fig. 2e and Additional file 2: Figure S7). Extensive organelle and cell swelling, cytoplasmic vacuolation were observed in resibufogenin-treated SW480 and HCT116. Transmission electron microscope provided further necrotic evidence of increased cell volume, dilatation of the nuclear membrane and condensation of chromatin in cells (Fig. 2f) and tissues (Fig. 2g) treated with resibufogenin and parthenolide. Therefore, resibufogenin induced cell death can be mainly classified as necrosis in both in vivo and in vitro tests.

**Fig. 3:**
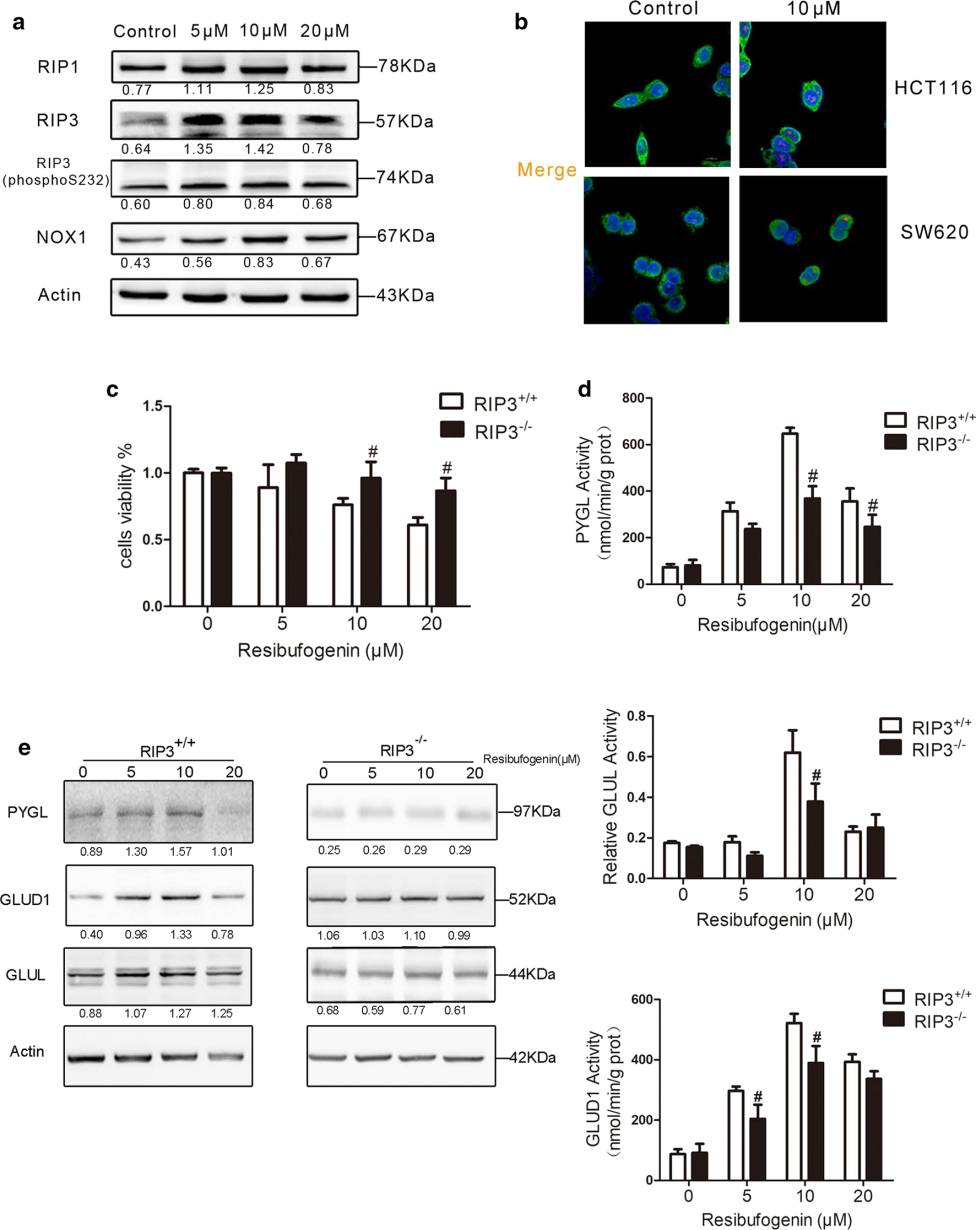
Resibufogenin induces RIP3-dependent necroptosis through activating PYGL, GLUL and GLUD1. **a** Immunoblot analysis of necrotic proteins were determined after 24 h of resibufogenin treatment. Level of Bax, P53, and PGAM5 in cytosol and mitochondrial fractions from HCT116 cells treated with resibufogenin at the indicated concentrations for 24 h were evaluated by western blot analysis. β-Actin was used as a loading control. See also Additional file 1 and Additional file 2: Figures S7, S8. The gray value of each stripe has been calculated using quantity one software. **b** Confocal immunofluorescence of RIP3 (red, anti-RIP3) in the HCT116 and SW620 cells that were loaded with MitoTracker and DAPI (n = 3). ×1000 for all, scale bar = 1 μm. **c** *RIP3*^+*/*+^and *RIP3*^−*/*−^ cells were treated with resibufogenin for 24 h. cells viability was measured by MTT assay (n = 6). See also Additional file 1 and Additional file 2: Figure S9. **d** The activity of three energy metabolism enzymes in the two cell lines (n = 3). **e** The protein expression of three energy—metabolizing enzymes in two cell lines. Data represent mean ± SEM, ^**#**^*P* < 0.05, *P < 0.01, as determined by one-way ANOVA followed by Tukey’s multiple comparison test

Cell damage caused by cell necrosis resulted in the release of enzymes into the culture medium [17]. As shown in Fig. 3, LDH activity was significantly elevated in cells treated with resibufogenin at 10 and 20 µM concentrations for 24 h (Fig. 2h). Intracellular ROS level was also increased in a dose-dependent manner in resibufogenin treated cells (Fig. 2i and Additional file 2: Figure S8). To associate ROS formation with resibufogenin-induced cell death, the effects of an antioxidant, l-NAC (l-*N*-acetyl-cysteine) on cell death were evaluated. l-NAC substantially enhanced the cell viability in HCT116 cells treated with resibufogenin (Fig. 2j), suggesting that ROS is the causative agent which leads to necrotic cell death. Together, these data indicates that resibufogenin induces cell death through ROS generation.

### Resibufogenin induces RIP3-dependent necroptosis through activating PYGL, GLUL and GLUD1

The nomenclature committee on cell death recommended that ‘necroptosis’ can be used to indicate the receptor-interacting protein kinase 1 (RIP1)- and/or RIP3-dependent regulated necrosis [18]. Western blotting showed that RIP3 was significantly elevated by resibufogenin while RIP-1 was only mildly upregulated (Fig. 3a). Immunofluorescence confirmed the upregulation of RIP3 in both HCT116 and SW480 cells (Fig. 3b). Quantitative real-time PCR indicated that the transcription of RIP3 increased to about 2–4 times in resibufogenin-treated HCT116 and SW620 cells, respectively (Additional file 1, Additional file 2: Figure S10).

The role of RIP3 in resibufogenin-induced cell death was further investigated with wild-type and *RIP3*^−*/*−^ mouse embryo fibroblasts (MEFs) [19]. As expected, the viability of *RIP3*^−*/*−^ cells was higher than that of corresponding wild type cells upon treatment with resibufogenin 10 and 20 μM for 24 h (Fig. 3c and Additional file 2: Figure S11). The activation of three key enzymes in energy metabolism, including glycogen phosphorylase (PYGL), glutamine synthetase (GLUL), and glutamate dehydrogenase (GLUDl) are involved in RIP3-mediated necroptosis [19, 20]. By measuring activity of PYGL, GLUD1 and GLUL in wild-type and *RIP3*^−*/*−^ MEFs before and after resibufogenin treatment, we found that RIP3 was required for resibufogenin-increased PYGL, GLUD1 and GLUL activity. The expression of PYGL, GLUD1 and GLUL were significantly increased in wild type cells but not in *RIP3*^−*/*−^ cells after treatment with resibufogenin (Fig. 3d). These results suggest that RIP3 is essential for resibufogenin induced necroptosis.

### Resibufogenin activates RIP3, PYGL, GLUD1 and GLUL in vivo

**Fig. 4:**
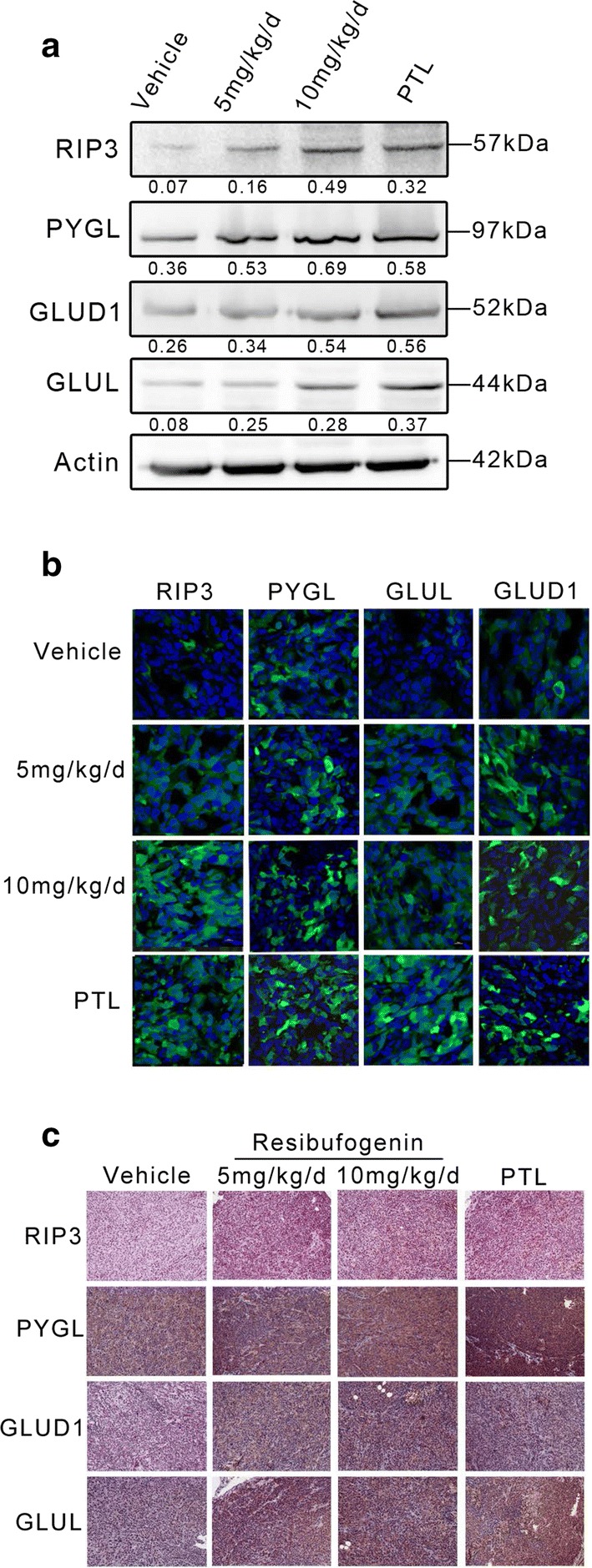
The expression of necroptosis protein in resibufogenin-, and parthenolide-treated mice. **a** RIP3, PYGL, GLUD1 and GLUL in tumor tissue lysates from vehicle-, resibufogenin-, and parthenolide-treated mice were detected by western blot analysis (n = 3). **b** Tumors were excised and processed for immunostaining with RIP3 (green, anti-RIP3), PYGL (green, anti-PYGL), GLUD1 (green, anti-GLUD1), GLUL (green, anti-GLUL) and DAPI (blue), and fluorescent images were obtained by Confocal, ×400 for all, scale bar = 100 μm. See also Additional file 1 and Additional file 2: Figure S10. **c** Immunohistochemical staining of RIP3, PYGL, GLUD1 and GLUL expression in tumor tissues. ×200 for all, scale bar = 100 μm

Western blot analysis in tissues from xenografts showed remarkable increase in levels of PYGL, GLUD1 and GLUL as well as increase of RIP3 in resibufogenin-treated mice compared with the control group (Fig. 4a). Immunostaining of histological sections also showed that resibufogenin stimulated the expression of RIP3, PYGL, GLUD1 and GLUL (Fig. 4b, c) in the tumor tissues. These in vivo data suggested that resibufogenin inhibits tumor growth through inducing RIP3-mediated activation of PYGL, GLUD1 and GLUL to induce necroptosis in CRC cells.

### Resibufogenin suppresses liver metastasis of CRC

**Fig. 5:**
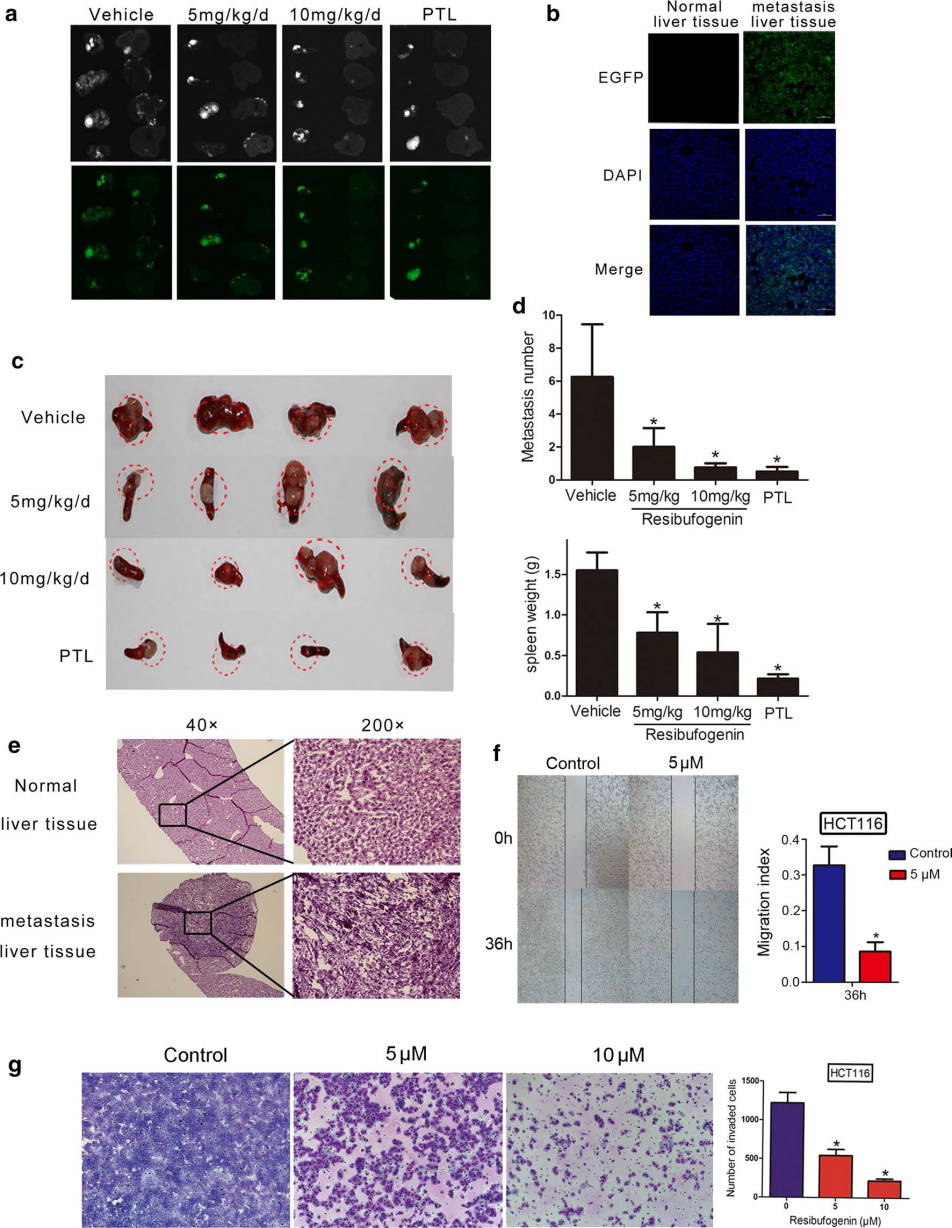
Resibufogenin suppresses metastasis by inhibiting EMT. **a** The fluorescent expression of spleen and liver was detected by imaging system. **b** The fluorescence expression of MC38-eGFP cells in the vehicle group was observed by confocal. ×200 for all, scale bar = 100 μm. **c** The picture of spleen tumor tissue in mice. **d** Bar graphs at the above show spleen weight from the MC38-eGFP mice. Bar graphs at the bottom show the percentages of metastasis number in mice hepatic tissue. *P < 0.01 as determined by one-way ANOVA followed by Tukey’s multiple comparison test. **e** HE staining of the difference between normal liver tissue and metastasis liver tissue. ×40,200 for all. F. Wound scratch assay was used to detect the migration ability of HCT116 cells after dosing representative micrographs from each condition. Bars represent migration distance compare to control group. *P < 0.01 as determined by one-way ANOVA. **g** Data of transwell assay for HCT116 cells. The cells were counted under a microscope in five randomly selected fields. Bars represent the number of cells invaded after dosing. The results were reproduced in three independent experiments. Date represent mean ± SEM. *P < 0.01 as determined by one-way ANOVA

To document the anti-metastatic action of resibufogenin in vivo, MC38, a colon adenocarcinoma cell line derived from C57BL/6 was injected beneath the splenic capsule of mice to produce liver metastases [19–22]. In vivo fluorescence imaging provided direct evidence that resibufogenin significantly reduced the metastatic foci in liver (Fig. 5a–c). Both the number and the size of metastatic foci were decreased by resibufogenin. Immunohistology provided further evidence of inhibiting liver metastasis by resibufogenin (Fig. 5e). To better understand the anti-metastasis effect of resibufogenin, cell invasion and cell migration was investigated by transwell migration assays and wound-healing, respectively. Resibufogenin dramatically reduced cell invasion in a dose-dependent manner as compared to vehicle group. It also significantly reduced cell migration at 36 h (*P* < 0.01, Fig. 5f, g). Transwell migration assay showed that the cell motility of *RIP3*^−*/*−^ cells were significantly higher than that of the wild type cells (Additional file 2: Figure S13). Resibufogenin also disrupted epithelial-mesenchymal transition (EMT) by increasing epithelial markers ZO-1 and E-cadherin and decreasing the expression of fibronectin, vimentin and Snail (Additional file 2: Figure S14) [23, 24]. Resibufogenin significantly reduced the cell migration in wild type MEFs as compared to *RIP3*^−*/*−^ cells, suggesting that resibufogenin suppress liver metastasis by inhibiting cell invasion and migration in a RIP3-dependent manner.

### Resibufogenin RIP3-dependently suppresses heterotropic CRC growth

**Fig. 6:**
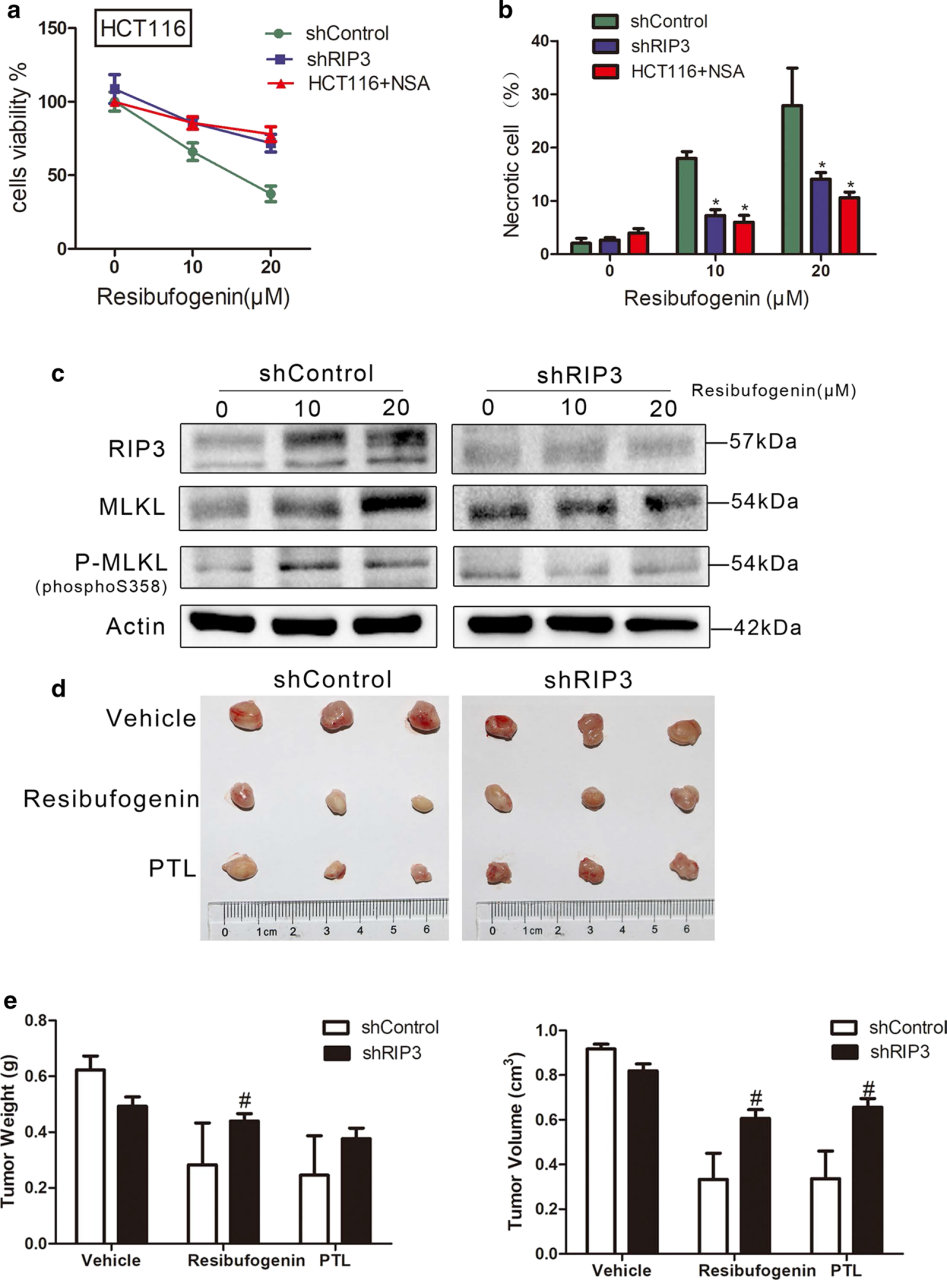
Resibufogenin RIP3-dependently suppresses heterotropic CRC growth. **a** HCT116 Cells were pretreated with NSA (1 μM) for 8 h prior to a 24 h treatment with resibufogenin. HCT116 + NSA, HCT116 shControl and HCT116 shRIP3 cells viability was measured by MTT assay (n = 6) **b** and the proportion of PI-positive cells was analyzed by flow cytometry (n = 3). **c** Western blotting experiments for RIP3, MLKL, P-MLKL (S358) were performed with the cell lysates obtained after resibufogenin treatment in HCT116 shControl and HCT116 shRIP3 cells. **d** The picture of colon tumor tissue in mice. **e** Isolated tumor size and tumor weight from the HCT116 shControl and HCT116 shRIP3 mice heterotropic CRC model

To further confirm resibufogenin-induced RIP3-dependent cell death in colorectal carcinoma, HCT116 cells were stably transduced with lentivirus carrying RIP3 short hairpin RNA (shRNA) that display more than 80% reduced expression of RIP3 protein (Additional file 1, Additional file 2: Figure S15). The lowered viability induced by resibufogenin was significantly rescued by RIP3-knockdown (Fig. 6a). Furthermore, resibufogenin induced necrotic cell deaths were neutralized by RIP3-knockdown and MLKL inhibitor necrosulfonamide (NSA), suggested the pivotal roles of RIP3 and MLKL in necrosis execution (Fig. 6b and Additional file 2: Figure S16).

The expression of RIP3, MLKL and phosphorylation of MLKL were upregulated by resibufogenin treatment. RIP3 knockdown significantly inactivated MLKL by lowering its phosphorylation at Serine 358 (Fig. 6c).

To explore the role of RIP3-dependent necroptosis in CRC progression, xenografts derived from RIP3 knockdown and control cells were treated with resibufogenin. The tumor volume and tumor weight were significantly by inhibited by resibufogenin. However, the tumor-suppressing effects of resibufogenin were abrogated by RIP3 knockdown, suggested that RIP3-dependent necroptosis is essential for the antineoplastic effects of resibufogenin (Fig. 6d, e).

**Fig. 7:**
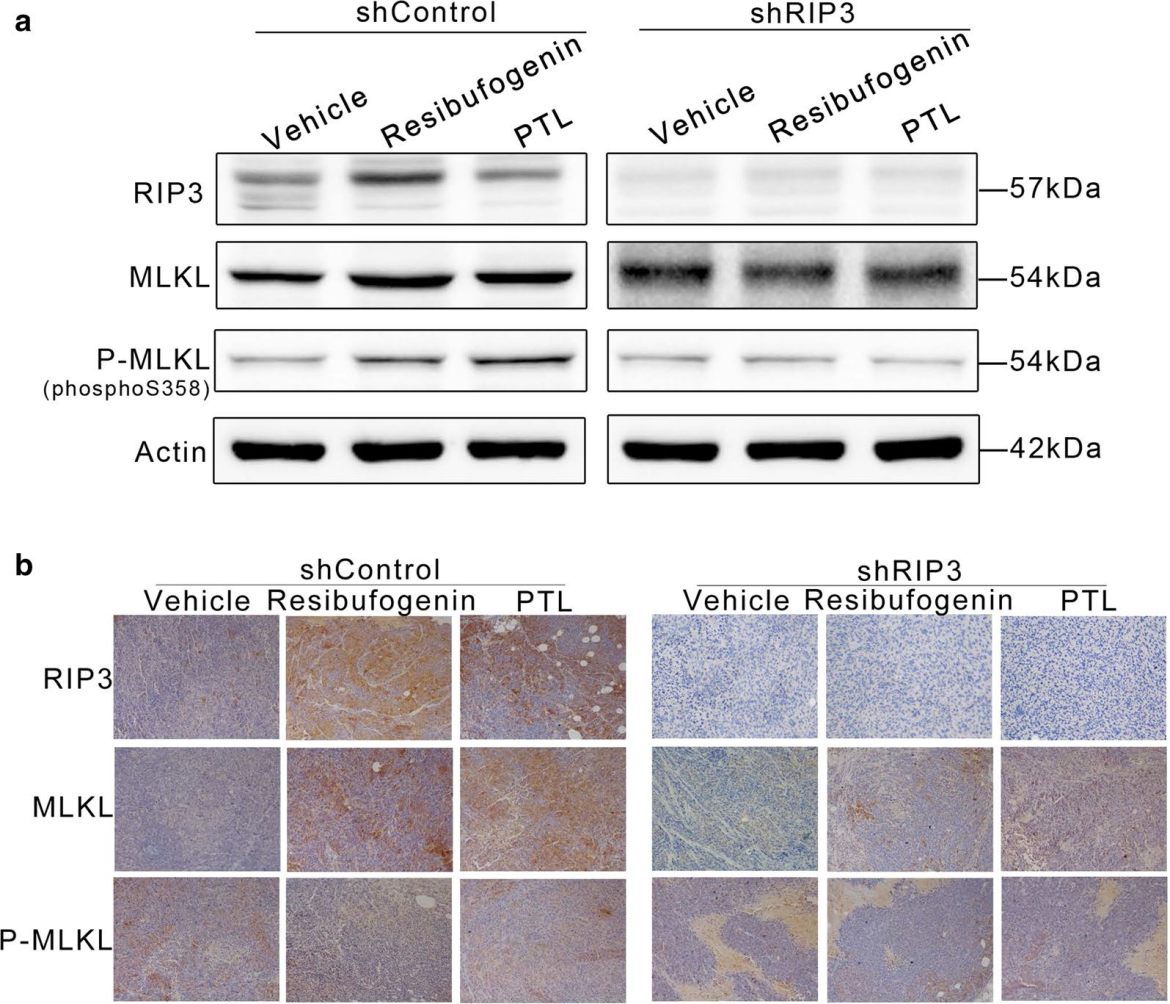
The expression of necroptosis protein in tumor tissue. **a** RIP3, MLKL, P-MLKL (S358) in tumor tissue lysates from vehicle-, resibufogenin-, and PTL-treated mice were detected by western blot analysis. **b** IHC analysis of the expression of protein in tumor tissue from vehicle-, resibufogenin-, and PTL-treated mice. ×200 for all, scale bar = 100 μm

The expression of RIP3 and MLKL were elevated in resibufogenin treated shControl tumor tissues but not in RIP3-knockdown tumors. MLKL phosphorylation was observed as expected in resibufogenin treated tumors but not in resibufogenin (Fig. 7a). The IHC staining provided consolidated data resibufogenin activated the expression of RIP3 and stimulated the phosphorylation of MLKL (Fig. 7b). These in vivo data suggest that resibufogenin inhibited the growth of CRC through necroptosis which depended on the activation of RIP3 and MLKL.

## Discussion

For a long time, necrosis has been considered as a passive process which is only an accidental cell death mechanism. Hence, it was merely defined by the absence of morphological traits of apoptosis or autophagy. It is now clear that necrosis can occur in a regulated manner. According to its specific nomenclature, necroptosis can be defined as RIP1- and/or RIP3-dependent regulated necrosis [18]. Resibufogenin-induced cell death is insensitive to zVAD, suggest it is not caspase-dependent apoptosis. Transmission electron microscopy provides the direct evidence of resibufogenin-induced necrosis. Resibufogenin increases the transcription of RIP3 at the mRNA level. It also promotes RIP3 expression and phosphorylation. Importantly, resibufogenin-induced necrosis can be significantly abrogated in *RIP3*^−*/*−^ MEF and RIP3 knockdown CRC cells. So, resibufogenin induced necrosis can be defined as RIP3-dependent necroptosis. Resibufogenin is a detectable component of “cinobufacini injection” which is permitted for clinical administration by China food and drug administration [25]. Further, the cytotoxic effects of resibufogenin in CRC cells are stronger than normal intestinal epithelial cells. So, inducing necroptosis by resibugogenin is an alternative mechanism that can be exploited to kill tumor cells [18].

Parthenolide markedly triggering cell necrosis by inducing ROS generation and mitochondrial dysfunction in breast cancer cells [15]. Parthenolide interacted with the cell membrane constituents that lead to the membrane rupture and induce primary necrosis [26], and thus was chosen as a necrotic inducer in the in vivo test. Both parthenolide and resibufogenin suppress CRC growth in vivo and in vitro by causing necroptosis. The activation of RIP3 phosphorylated MLKL at S358 and derived its oligomerization which directly disrupted membrane integrity during necroptosis [26, 27]. So, MLKL is phosphorylated by RIP3 upon resibufogenin treatment and recruited to form necrosome through its interaction with RIP3 [28].

PYGL catalyzes the degradation of glycogen by releasing glucose-1-phosphate and therefore plays a key role in utilizing glycogen reserves as an energy source. GLUL is a cytosolic enzyme that catalyzes the condensation of glutamate and ammonia to form glutamine, whereas GLUD1 is found in the mitochondrial matrix and converts glutamate to α-ketoglutarate [29]. RIP3 might interact with and activate PYGL, GLUL and GLUD1 upon resibufogenin treatment, thereby increasing their enzymatic activity and promoting a considerable metabolic burst [19, 30]. This in turn propagates the overgeneration of ROS, thereby favoring mitochondrial dysfunction and necroptosis [30]. So, resibufogenin-induced necroptosis at least partly occurs through increasing ROS production in a RIP3-dependent manner as a consequence of energy metabolism.

RIP3 is significantly decreased in CRC tissues as compared to adjacent normal colon tissues. Its downregulation impairs the cancer cells’ response to necroptosis triggers [31]. Our results are consistent with the report that shikonin inhibits cancer cell metastasis by inducing RIP1/3 expression and promoting necroptosis [32]. However, the role of necroptosis in cancer remains controversial. Emerging research supports the idea that necroptosis promotes the proliferation and metastasis of living cancer cells through induction of inflammation and ROS. The net effect of necroptosis on cancer progression might depend on the cancer stage and release contents of dead cells [33]. Our orthotopic CRC tests suggested that resibufogenin suppresses CRC growth and metastasis by inducing necroptosis. It is worth noting that the activation of RIP3, PYGL, GLUL and GLUD1 can not only be observed in cells in vitro, but can also be induced in vivo in mice treated with resibufogenin. RIP3 is not only a molecular switch for necroptosis, but also a hub governing the cell’s metabolic state [29, 34, 35].

## Conclusions

Resibufogenin retards tumor growth and metastasis by activating RIP3 and subsequently phosphorylating MLKL which lead to necroptosis. Resibufogenin-induced necroptosis can be a dormant killer to be harnessed to achieve the ultimate goal of killing tumor cells.

## Additional files

**Additional file 1.** Additional tables.

**Additional file 2.** Additional figures.

## Abbreviations

EMT: epithelial–mesenchymal transition
GLUD1: glutamate dehydrogenase1
GLUL: glutamate–ammonialigase
PYGL: glycogen phosphorylase
RIP1: receptor-interacting protein kinase 1
RIP3: receptor-interacting protein kinase 3
LDH: lactate dehydrogenase
MEFs: mouse embryo fibroblasts
ROS: reactive oxygen species
z-VAD-FMK: *N*-benzyloxycarbonyl-Val-Ala-Asp-fluoromethylketone
