# Supplementary material for: Resibufogenin suppresses colorectal cancer growth and metastasis through RIP3-mediated necroptosis
Source: J Transl Med. 2018 Jul 20;16:201. doi: 10.1186/s12967-018-1580-x (PMC6053767; doi:10.1186/s12967-018-1580-x)
Supplement: Supplementary file 1 — Additional file 1. Additional tables. [file 12967_2018_1580_MOESM1_ESM.docx]

**Additional materials (Tables of Confidence intervals)**

Table S1. Statistical analysis of HCT116 cells double stained with Annexin V & PI in 24h

| Concentration | ‾*x*±*S* | *95%CI* | *F* | *P* | N |
| --- | --- | --- | --- | --- | --- |
| 0 | 0.03±0.02 | (0.00,0.07) | 22.79 | <0.01^*^ | 3 |
| 5 | 0.18±0.06 | (0.03,0.33) |  |  | 3 |
| 10 | 0.35±0.09 | (0.13,0.57) |  |  | 3 |
| 20 | 0.72±0.18 | (0.26,1.17) |  |  | 3 |

*P* < 0.05^#^，*P* < 0.01^*^

Table S2. Statistical analysis of SW480 cells double stained with Annexin V & PI in 24h

| Concentration | ‾*x±S* | *95%CI* | *χ^2^* | *P* | N |
| --- | --- | --- | --- | --- | --- |
| 0 | 0.03±0.01 | (0.00,0.06) | 9.08 | 0.028^△#^ | 3 |
| 5 | 0.12±0.10 | (-0.13,0.37) |  |  | 3 |
| 10 | 0.39±0.11 | (0.12,0.65) |  |  | 3 |
| 20 | 0.60±0.19 | (0.14,1.07) |  |  | 3 |

^△^Heterogeneity of variance, rank sum test with Kruskal-Wallis was performed for analysis among groups. *P* < 0.05^#^，*P* < 0.01^*^

Table S3. Statistical analysis of SW480 cells double stained with Annexin V & 7AAD detected by FCM in 24h

| Concentration | ‾*x±S* | *95%CI* | *F* | *P* | N |
| --- | --- | --- | --- | --- | --- |
| 0 | 2.35±0.74 | (0.52,4.20) | 95.97 | <0.01^*^ | 3 |
| 5 | 5.48±2.58 | (-0.93,11.88) |  |  | 3 |
| 10 | 24.85±5.89 | (10.22,39.48) |  |  | 3 |
| 15 | 54.74±4.61 | (43.29,66.19) |  |  | 3 |
| 20 | 53.32±5.98 | (38.46,68.17) |  |  | 3 |

*P* < 0.05^#^，*P* < 0.01^*^

Table S4. Statistical analysis of SW480 cells double stained with Annexin V & 7AAD detected by FCM in 48h

| Concentration | ‾*x±S* | *95%CI* | *F* | *P* | N |
| --- | --- | --- | --- | --- | --- |
| 0 | 2.89±0.93 | (0.57,5.21) | 114.40 | <0.01^*^ | 3 |
| 5 | 16.24±3.08 | (8.60,23.89) |  |  | 3 |
| 10 | 43.83±6.08 | (28.71,58.95) |  |  | 3 |
| 15 | 62.02±8.34 | (41.29,82.74) |  |  | 3 |
| 20 | 80.15±3.96 | (70.31,89.99) |  |  | 3 |

*P* < 0.05^#^，*P* < 0.01^*^

Table S5. Statistical analysis of HCT116 cells double stained with Annexin V & 7AAD detected by FCM in 24h

| Concentration | ‾*x±S* | *95%CI* | *χ^2^* | *P* | N |
| --- | --- | --- | --- | --- | --- |
| 0 | 1.79±2.02 | (-3.23,6.81) | 11.83 | 0.019^△#^ | 3 |
| 5 | 4.90±2.55 | (-1.43,11.24) |  |  | 3 |
| 10 | 4.10±1.47 | (0.46,7.74) |  |  | 3 |
| 15 | 32.41±12.48 | (1.39,63.42) |  |  | 3 |
| 20 | 59.98±7.55 | (41.24,78.73) |  |  | 3 |

^△^Heterogeneity of variance, rank sum test with Kruskal-Wallis was performed for analysis among groups. *P* < 0.05^#^，*P* < 0.01^*^

Table S6.Statistical analysis of HCT116 cells double stained with Annexin V & 7AAD detected by FCM in 48h

| Concentration | ‾*x±S* | *95%CI* | *F* | *P* | N |
| --- | --- | --- | --- | --- | --- |
| 0 | 2.91±1.26 | (-0.21,6.04) | 106.19 | <0.01^*^ | 3 |
| 5 | 5.91±1.91 | (1.17,10.65) |  |  | 3 |
| 10 | 13.42±3.04 | (5.88,20.97) |  |  | 3 |
| 15 | 47.44±7.21 | (29.55,65.33) |  |  | 3 |
| 20 | 66.508±6.80 | (49.61,83.39) |  |  | 3 |

*P* < 0.05^#^，*P* < 0.01^*^

Table S7. Statistical analysis of the activity of PYGL

| Concentration | +/+ | | -/- | | *F* | *P* | N |
| --- | --- | --- | --- | --- | --- | --- | --- |
|  | ‾*x±S* | *95%CI* | ‾*x±S* | *95%CI* |  |  |  |
| 0 | 72.32±21.96 | (49.27,95.36) | 80.30±37.20 | (41.26,119.34) | 4.36 | 0.063 | 6 |
| 5 | 313.42±58.18 | (252.36,374.47) | 236.89±35.44 | (199.70,274.08) | 2.55 | 0.141 | 6 |
| 10 | 647.17±39.06 | (606.18,688.17) | 368.45±81.87 | (282.53,454.36) | 8.26 | 0.017^#^ | 6 |
| 20 | 355.49±87.25 | (263.92,447.05) | 280.07±38.79 | (239.37,320.78) | 10.07 | 0.010^#^ | 6 |

*P* < 0.05^#^，*P* < 0.01^*^

Table S8. Statistical analysis of the activity of GLUD1

| Concentration | +/+ | | -/- | | *F* | *P* | N |
| --- | --- | --- | --- | --- | --- | --- | --- |
|  | ‾*x±S* | *95%CI* | ‾*x±S* | *95%CI* |  |  |  |
| 0 | 87.38±24.24 | (61.95,112.81) | 91.71±45.95 | (43.49,139.93) | 2.69 | 0.18 | 6 |
| 5 | 296.88±22.34 | (273.44,320.33) | 253.96±70.29 | (180.20,327.73) | 10.73 | 0.008^#^ | 6 |
| 10 | 521.92±47.82 | (471.73,572.10) | 389.90±86.24 | (299.40,480.40) | 5.54 | 0.04* | 6 |
| 20 | 392.98±39.43 | (351.60,434.36) | 292.80±49.55 | (240.81,344.80) | 0.43 | 0.527 | 6 |

*P* < 0.05^#^，*P* < 0.01^*^

Table S9. Statistical analysis of the activity of GLUL

| Concentration | +/+ | | -/- | | *F* | *P* | N |
| --- | --- | --- | --- | --- | --- | --- | --- |
|  | ‾*x±S* | *95%CI* | ‾*x±S* | *95%CI* |  |  |  |
| 0 | 0.18±0.02 | (0.17,0.20) | 0.16±0.02 | (0.15,0.18) | 0.22 | 0.65 | 6 |
| 5 | 0.19±0.05 | (0.14,0.24) | 0.12±0.03 | (0.09,0.15) | 1.31 | 0.28 | 6 |
| 10 | 0.63±0.17 | (0.45,0.81) | 0.49±0.05 | (0.44,0.54) | 9.67 | 0.011* | 6 |
| 20 | 0.24±0.04 | (0.20,0.28) | 0.26±0.10 | (0.16,0.36) | 3.29 | 0.08 | 6 |

*P* < 0.05^#^，*P* < 0.01^*^

Table S10. Statistical analysis on ROS level

| Concentration | ‾*x*±*S* | *95%CI* | *F* | *P* | N |
| --- | --- | --- | --- | --- | --- |
| 0 | 24.09±5.91 | (9.40,38.78) | 70.04 | <0.01^*^ | 3 |
| 5 | 47.17±7.00 | (29.78,64.56) |  |  | 3 |
| 10 | 70.00±5.59 | (56.13,83.88) |  |  | 3 |
| 20 | 89.72±4.78 | (77.85,101.59) |  |  | 3 |

*P* < 0.05^#^，*P* < 0.01^*^

Table S11. Statistical analysis on LDH level

| Concentration | ‾*x±S* | *95%CI* | *χ^2^* | *P* | N |
| --- | --- | --- | --- | --- | --- |
| 0 | 91.14±9.53 | (67.48,114.80) | 13.23 | 0.010^*^ | 3 |
| 1 | 128.16±19.10 | (80.71,175.61) |  |  | 3 |
| 5 | 207.24±20.58 | (156.13,258.36) |  |  | 3 |
| 10 | 259.79±51.79 | (131.13,388.45) |  |  | 3 |
| 20 | 371.36±23.71 | (312.46,430.27) |  |  | 3 |

^△^Heterogeneity of variance, rank sum test with Kruskal-Wallis was performed for analysis among groups. *P* < 0.05^*^，*P* < 0.01^#^
