# Supplementary material for: Resibufogenin suppresses colorectal cancer growth and metastasis through RIP3-mediated necroptosis
Source: J Transl Med. 2018 Jul 20;16:201. doi: 10.1186/s12967-018-1580-x (PMC6053767; doi:10.1186/s12967-018-1580-x)
Supplement: Supplementary file 2 — Additional file 2. Additional figures. [file 12967_2018_1580_MOESM2_ESM.docx]

**Additional Information**


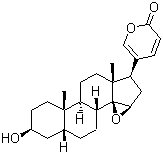


Figure S1. Chemical structural formula of resibufogenin


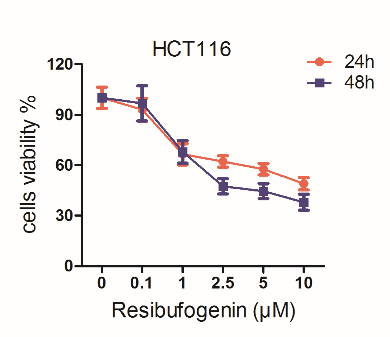

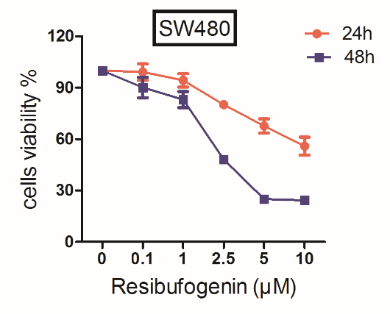

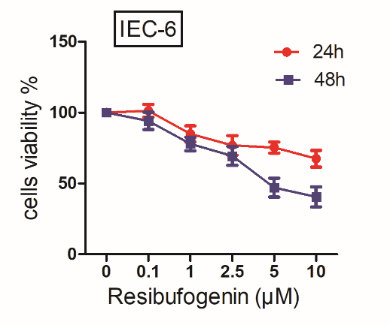


Figure S2. Effects of resibufogenin on proliferation of HCT116, SW480 and IEC-6.

SW480 and IEC-6 cells were exposed to either vehicle or 0.1–10 μM resibufogenin and incubated for 24 and 48 h. Cell viability was measured by MTT methods (n = 6 per group).


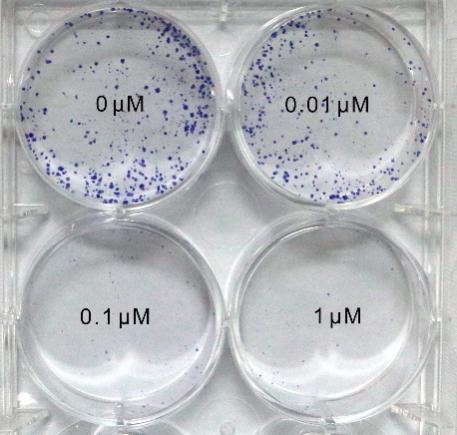


Figure S3. The effects of resibufogenin on clone formation in SW480 cells.

The image of plate cloning tests. Related to Fig. 2B.


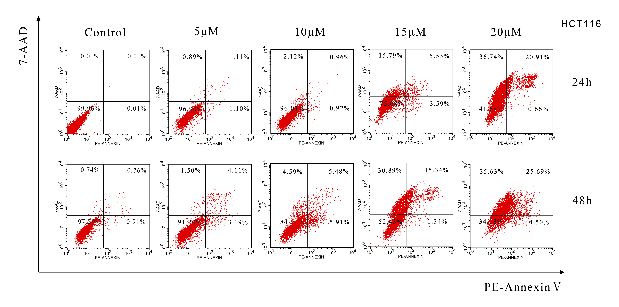

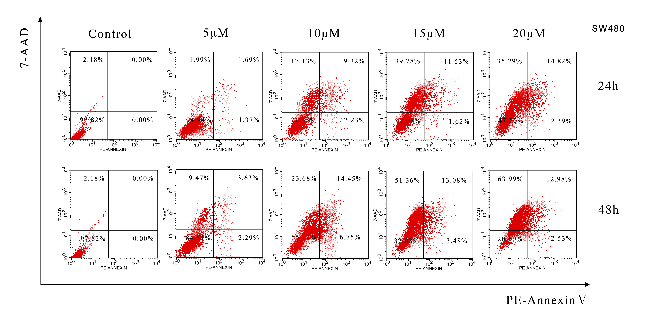


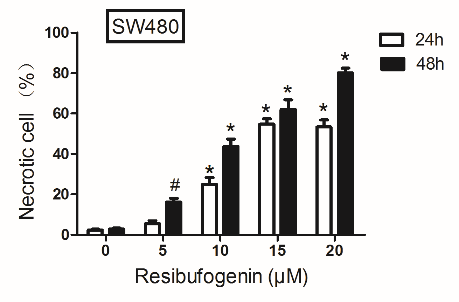


Figure S4. The effects of resibufogenin on cell death in HCT116 and SW480 cells.

HCT116, SW480 cells were treated with resibufogenin for different time, The necrotic cell was evaluated with flow cytometry after stained with PE-annexin V/7-amino-actinomycin D. The necrotic cell were quantitatively analyzed. Related to Figure 2C. **P*  < 0.01 indicate significant difference.


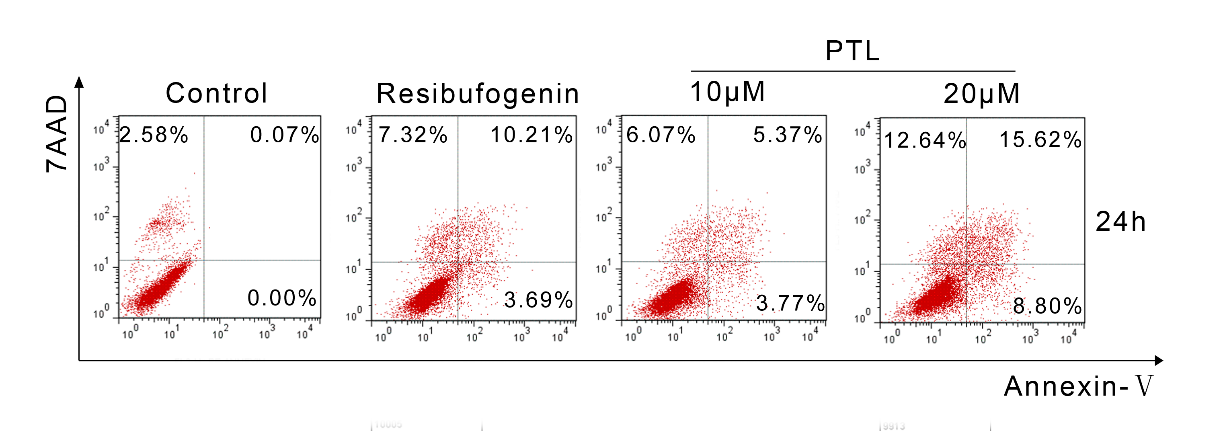


Figure S5. Necrosis of colon cancer cells treated with PTL detected by flow cytometry

The necrotic cell of HCT116 cells was detected by flow cytometry treated with PTL, resibufogenin as positive control. Related to Figure 2A.


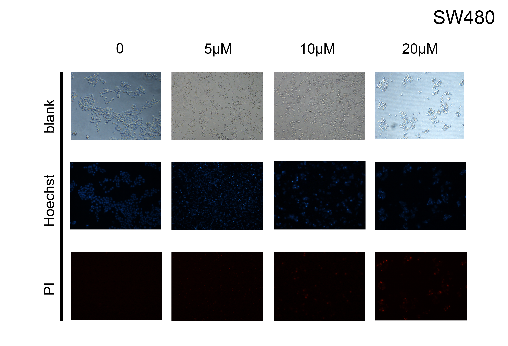

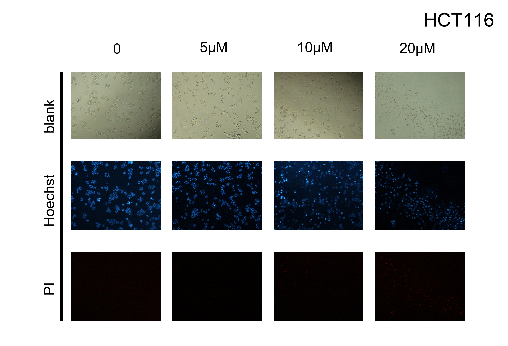


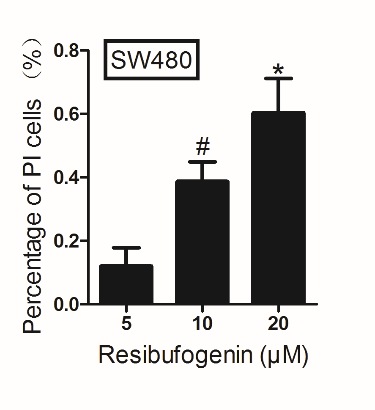


Figure S6. The effects of resibufogenin on cell death in HCT116 and SW480 cells.

HCT116, SW480 cells were incubated with resibufogenin for indicated time and stained with Hoechst33342 and PI for 15 min at room temperature.Images were analyzed. 100× for all. Related to Figure 2D. ^#^*P*  < 0.05,**P*  < 0.01 as determined by one-way ANOVA.


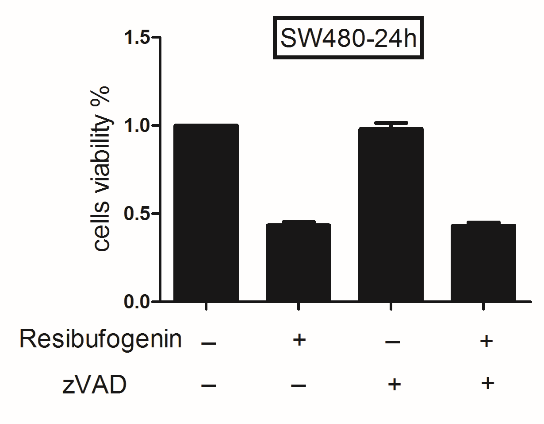

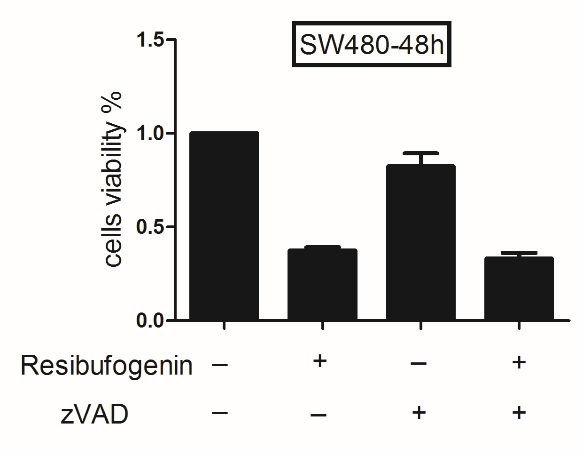


Figure S7. The effects of resibufogenin on cell viability with zVAD in SW480 cells.

SW480 were treated with resibufogenin and Z-VAD-fmk (20 μM). Cell viability was measured by MTT assay. Data represent mean ± SEM, n = 6. Related to Figure 2E.


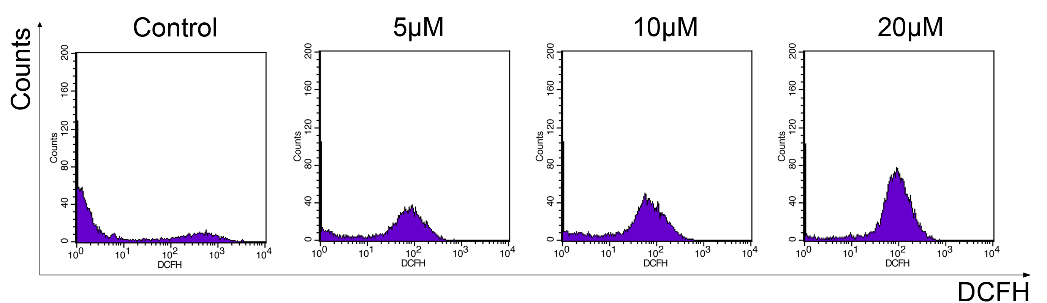


Figure S8. The effects of resibufogenin on ROS level in HCT116 cells.

HCT116 Cells were treated with resibufogenin at different concentrations (0, 5, 10, 20μM) for 24 hrs. ROS levels were detected by flow cytometry. Related to Figure 2I.

.
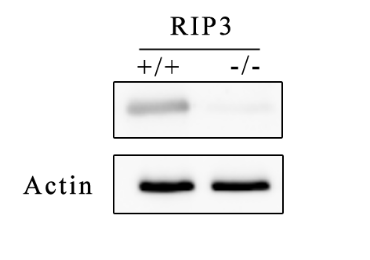

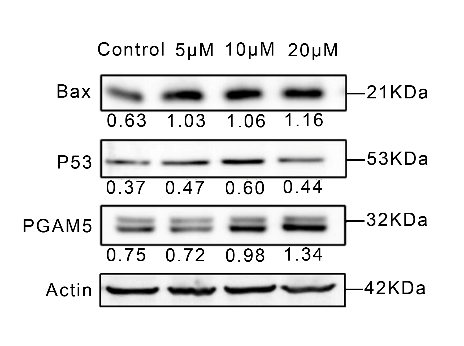


Figure S9. The effects of resibufogenin on expression of related proteins in HCT116 cells.

Immunoblot analysis of RIP3 in wild-type and *RIP3^-/-^* mouse embryo fibroblast (MEF) cells. Level of Bax, P53, and PGAM5 in cytosol and mitochondrial fractions from HCT116 cells treated with resibufogenin at the indicated concentrations for 24 h were evaluated by western blot analysis. The gray value of each stripe has been calculated using quantity one software.


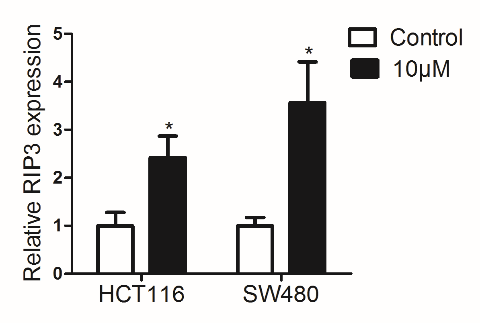


Figure S10. The effects of resibufogenin on RIP3 mRNA expression in HCT116 and SW480 cells.

Gene expression analysis of RIP3 of colon cells by RT-qPCR. The relative quantification value, fold difference, is expressed as 2^−ΔΔCt^. vs. NC. **P* < 0.01 as determined by Student’s t-test.


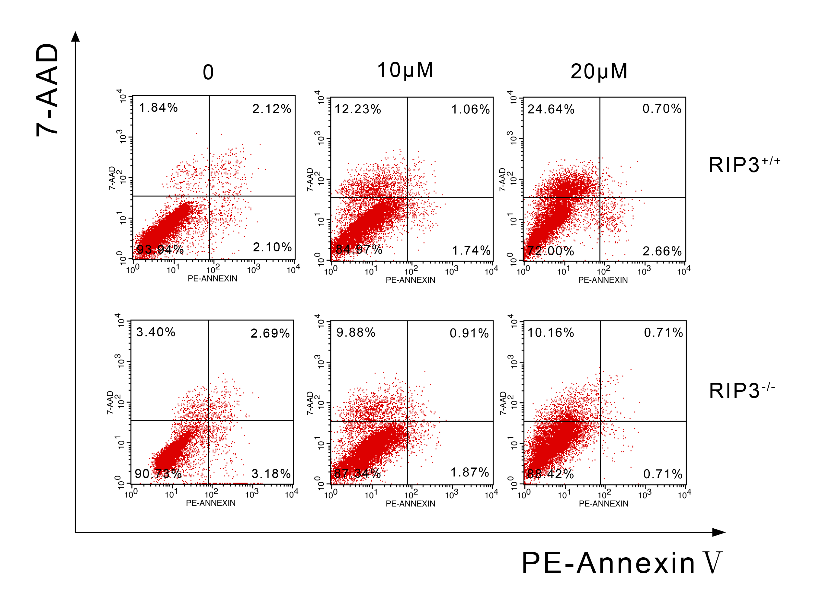

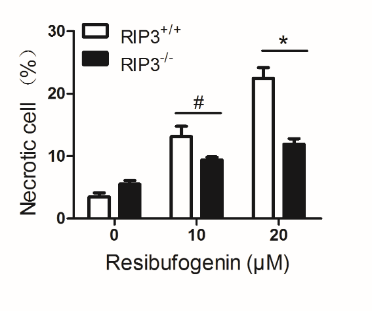


Figure S11. The effects of resibufogenin on cell death in *RIP3^+/+^RIP3^-/-^*cells.

The necrotic cell of *RIP3^+/+^RIP3^-/-^*cells was detected by flow cytometry. ^#^*P* < 0.05,**P*  < 0.01 as determined by one-way ANOVA.


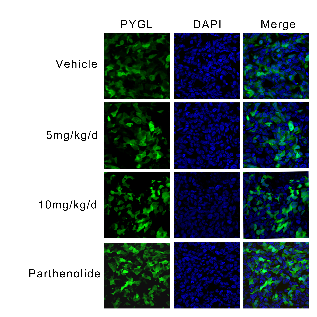

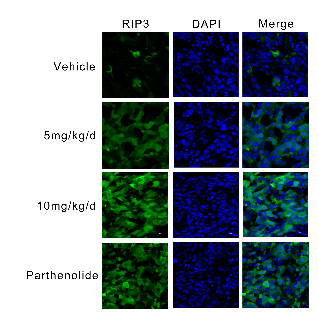

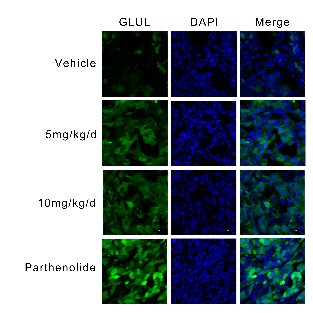

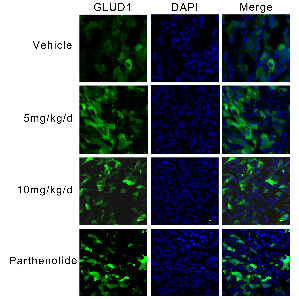
Figure S12. The effects of resibufogenin on three key enzymes in tumor tissues.

Immunofluorescence staining of RIP3 (green, anti-RIP3), PYGL (green, anti-PYGL), GLUD1 (green, anti-GLUD1), GLUL (green, anti-GLUL) expression in heterotropic CRC tumor tissues. 400× for all. scale bar = 100 μm. Related to Fig.4B.


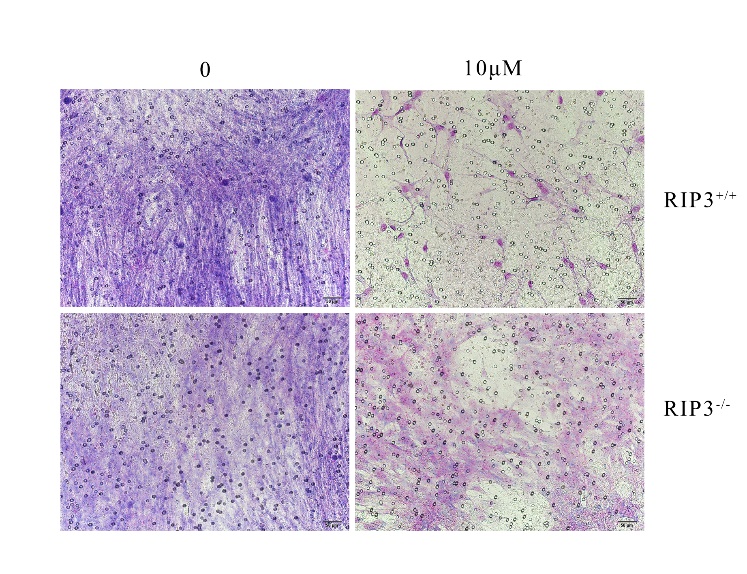


Figure S13. Data of transwell assay for *RIP3^+/+^* and *RIP3^-/-^* cells.

*RIP3^-/-^* cells were significantly higher than that of *RIP3^+/+^* cells on cell motility.


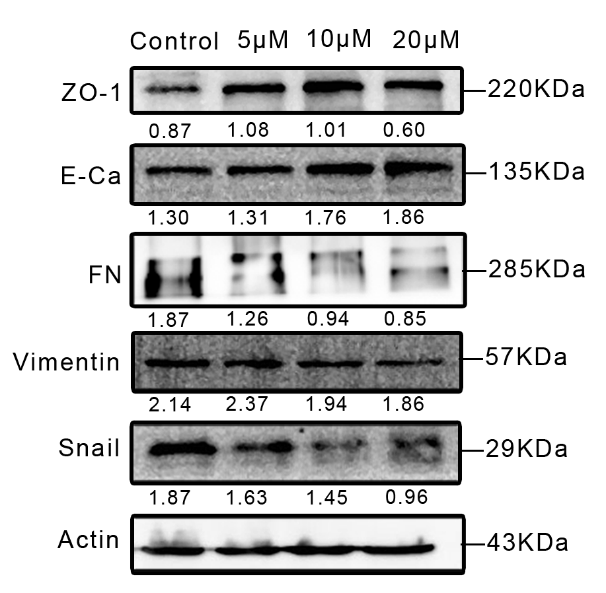


Figure S14. Total cell lysates were prepared for western blot analysis of EMT proteins.

The expression of epithelial markers ZO-1 and E-cadherin and the expression of fibronectin, vimentin and Snail were tested by western blot. The gray value of each stripe has been calculated using quantity one software.


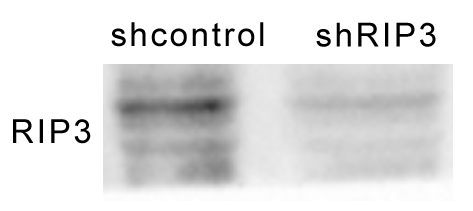


Figure S15. Western blot analysis of RIP3 expression in RIP3 knock down cells

HCT116 cells were stably transduced with lentivirus carrying RIP3 short hairpin RNA (shRNA) which analysed by western blot.


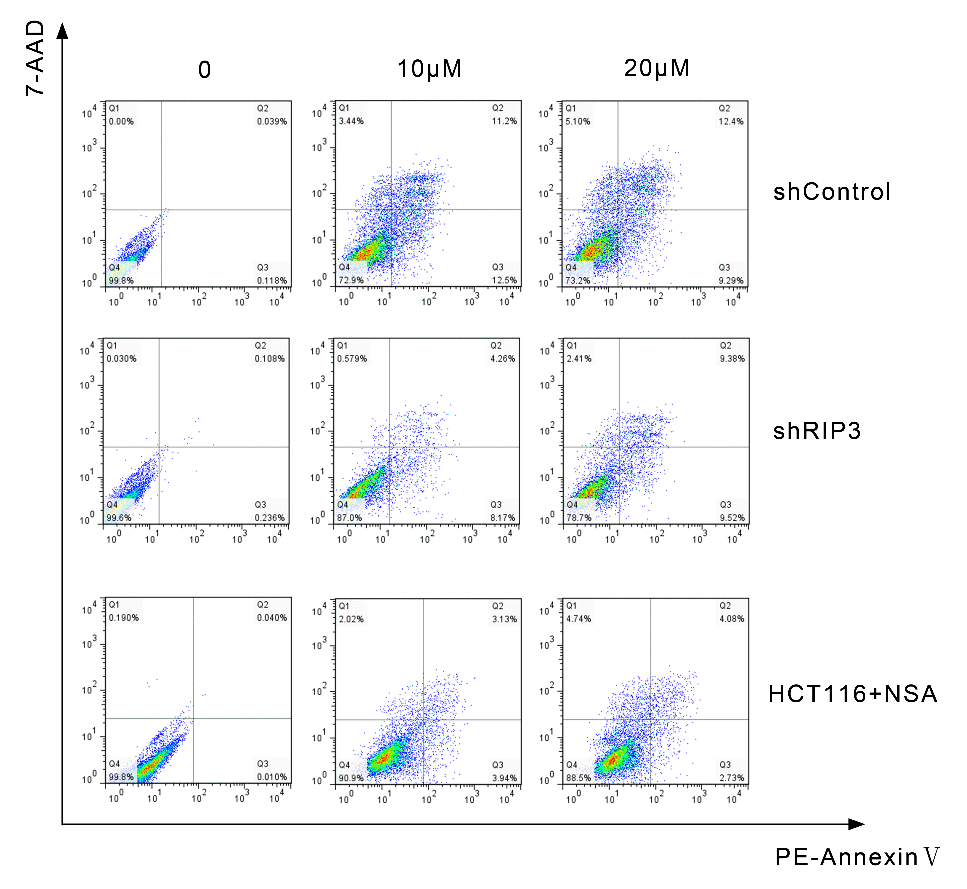


Figure S16. Necrosis of HCT116+NSA, HCT116 shControl and HCT116 shRIP3 cells treated with resibufogenin detected by flow cytometry.

HCT116 Cells were pretreated with NSA (1μM) for 8 h prior to a 24 h treatment with resibufogenin. HCT116+NSA, HCT116 shControl and HCT116 shRIP3 cells proportion of PI-positive cells were analyzed by flow cytometry (n=3). Related to Fig.6B.


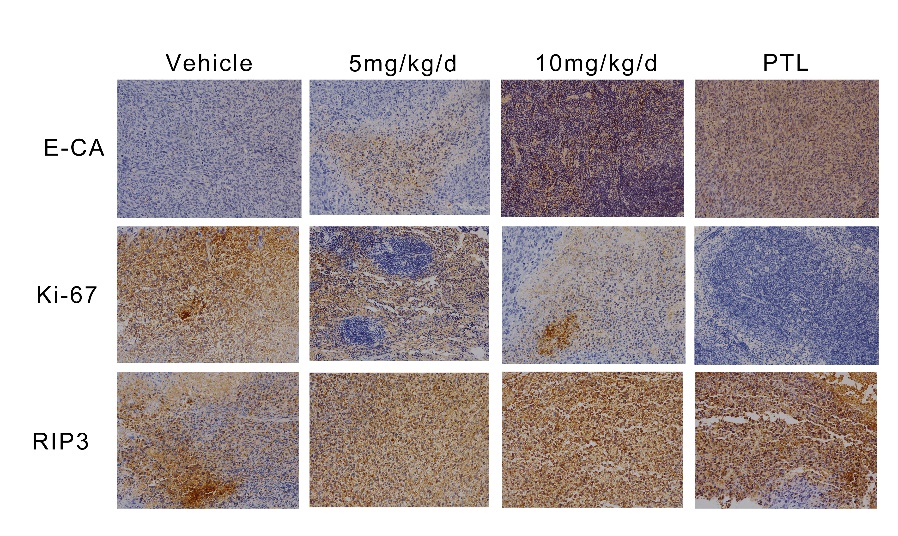


Figure S17. IHC analysis of the expression of E-cadherin, Ki-67 and RIP3 in MC38 mouse model.

Level of Ki-67, E-CA, RIP3 treated with resibufogenin and PTL were evaluated by IHC analysis. 200× for all, scale bar = 100 μm.

Statistical anylasis of HCT116 cells double stained with Annexin V & PI in 24h

| Concentration | ‾*x*±*S* | *95%CI* | *F* | *P* | N |
| --- | --- | --- | --- | --- | --- |
| 0 | 0.03±0.02 | (0.00,0.07) | 22.79 | <0.01^*^ | 3 |
| 5 | 0.18±0.06 | (0.03,0.33) |  |  | 3 |
| 10 | 0.35±0.09 | (0.13,0.57) |  |  | 3 |
| 20 | 0.72±0.18 | (0.26,1.17) |  |  | 3 |

*P* < 0.05^#^，*P* < 0.01^*^

Statistical anylasis of SW480 cells double stained with Annexin V & PI in 24h

| Concentration | ‾*x±S* | *95%CI* | *χ^2^* | *P* | N |
| --- | --- | --- | --- | --- | --- |
| 0 | 0.03±0.01 | (0.00,0.06) | 9.08 | 0.028^△#^ | 3 |
| 5 | 0.12±0.10 | (-0.13,0.37) |  |  | 3 |
| 10 | 0.39±0.11 | (0.12,0.65) |  |  | 3 |
| 20 | 0.60±0.19 | (0.14,1.07) |  |  | 3 |

^△^Heterogeneity of variance, rank sum test with Kruskal-Wallis was performed for analysis among groups. *P* < 0.05^#^，*P* < 0.01^*^

Statistical anylasis of SW480 cells double stained with Annexin V & 7AAD detected by FCM in 24h

| Concentration | ‾*x±S* | *95%CI* | *F* | *P* | N |
| --- | --- | --- | --- | --- | --- |
| 0 | 2.35±0.74 | (0.52,4.20) | 95.97 | <0.01^*^ | 3 |
| 5 | 5.48±2.58 | (-0.93,11.88) |  |  | 3 |
| 10 | 24.85±5.89 | (10.22,39.48) |  |  | 3 |
| 15 | 54.74±4.61 | (43.29,66.19) |  |  | 3 |
| 20 | 53.32±5.98 | (38.46,68.17) |  |  | 3 |

*P* < 0.05^#^，*P* < 0.01^*^

Statistical anylasis of SW480 cells double stained with Annexin V & 7AAD detected by FCM in 48h

| Concentration | ‾*x±S* | *95%CI* | *F* | *P* | N |
| --- | --- | --- | --- | --- | --- |
| 0 | 2.89±0.93 | (0.57,5.21) | 114.40 | <0.01^*^ | 3 |
| 5 | 16.24±3.08 | (8.60,23.89) |  |  | 3 |
| 10 | 43.83±6.08 | (28.71,58.95) |  |  | 3 |
| 15 | 62.02±8.34 | (41.29,82.74) |  |  | 3 |
| 20 | 80.15±3.96 | (70.31,89.99) |  |  | 3 |

*P* < 0.05^#^，*P* < 0.01^*^

Statistical anylasis of HCT116 cells double stained with Annexin V & 7AAD detected by FCM in 24h

| Concentration | ‾*x±S* | *95%CI* | *χ^2^* | *P* | N |
| --- | --- | --- | --- | --- | --- |
| 0 | 1.79±2.02 | (-3.23,6.81) | 11.83 | 0.019^△#^ | 3 |
| 5 | 4.90±2.55 | (-1.43,11.24) |  |  | 3 |
| 10 | 4.10±1.47 | (0.46,7.74) |  |  | 3 |
| 15 | 32.41±12.48 | (1.39,63.42) |  |  | 3 |
| 20 | 59.98±7.55 | (41.24,78.73) |  |  | 3 |

^△^Heterogeneity of variance, rank sum test with Kruskal-Wallis was performed for analysis among groups. *P* < 0.05^#^，*P* < 0.01^*^

Statistical anylasis of HCT116 cells double stained with Annexin V & 7AAD detected by FCM in 48h

| Concentration | ‾*x±S* | *95%CI* | *F* | *P* | N |
| --- | --- | --- | --- | --- | --- |
| 0 | 2.91±1.26 | (-0.21,6.04) | 106.19 | <0.01^*^ | 3 |
| 5 | 5.91±1.91 | (1.17,10.65) |  |  | 3 |
| 10 | 13.42±3.04 | (5.88,20.97) |  |  | 3 |
| 15 | 47.44±7.21 | (29.55,65.33) |  |  | 3 |
| 20 | 66.508±6.80 | (49.61,83.39) |  |  | 3 |

*P* < 0.05^#^，*P* < 0.01^*^

Statistical anylasis of the activity of PYGL

| Concentration | +/+ | | -/- | | *F* | *P* | N |
| --- | --- | --- | --- | --- | --- | --- | --- |
|  | ‾*x±S* | *95%CI* | ‾*x±S* | *95%CI* |  |  |  |
| 0 | 72.32±21.96 | (49.27,95.36) | 80.30±37.20 | (41.26,119.34) | 4.36 | 0.063 | 6 |
| 5 | 313.42±58.18 | (252.36,374.47) | 236.89±35.44 | (199.70,274.08) | 2.55 | 0.141 | 6 |
| 10 | 647.17±39.06 | (606.18,688.17) | 368.45±81.87 | (282.53,454.36) | 8.26 | 0.017^#^ | 6 |
| 20 | 355.49±87.25 | (263.92,447.05) | 280.07±38.79 | (239.37,320.78) | 10.07 | 0.010^#^ | 6 |

*P* < 0.05^#^，*P* < 0.01^*^

Statistical anylasis of the activity of GLUD1

| Concentration | +/+ | | -/- | | *F* | *P* | N |
| --- | --- | --- | --- | --- | --- | --- | --- |
|  | ‾*x±S* | *95%CI* | ‾*x±S* | *95%CI* |  |  |  |
| 0 | 87.38±24.24 | (61.95,112.81) | 91.71±45.95 | (43.49,139.93) | 2.69 | 0.18 | 6 |
| 5 | 296.88±22.34 | (273.44,320.33) | 253.96±70.29 | (180.20,327.73) | 10.73 | 0.008^#^ | 6 |
| 10 | 521.92±47.82 | (471.73,572.10) | 389.90±86.24 | (299.40,480.40) | 5.54 | 0.04* | 6 |
| 20 | 392.98±39.43 | (351.60,434.36) | 292.80±49.55 | (240.81,344.80) | 0.43 | 0.527 | 6 |

*P* < 0.05^#^，*P* < 0.01^*^

Statistical anylasis of the activity of GLUL

| Concentration | +/+ | | -/- | | *F* | *P* | N |
| --- | --- | --- | --- | --- | --- | --- | --- |
|  | ‾*x±S* | *95%CI* | ‾*x±S* | *95%CI* |  |  |  |
| 0 | 0.18±0.02 | (0.17,0.20) | 0.16±0.02 | (0.15,0.18) | 0.22 | 0.65 | 6 |
| 5 | 0.19±0.05 | (0.14,0.24) | 0.12±0.03 | (0.09,0.15) | 1.31 | 0.28 | 6 |
| 10 | 0.63±0.17 | (0.45,0.81) | 0.49±0.05 | (0.44,0.54) | 9.67 | 0.011* | 6 |
| 20 | 0.24±0.04 | (0.20,0.28) | 0.26±0.10 | (0.16,0.36) | 3.29 | 0.08 | 6 |

*P* < 0.05^#^，*P* < 0.01^*^

Statistical anylasis on ROS level

| Concentration | ‾*x*±*S* | *95%CI* | *F* | *P* | N |
| --- | --- | --- | --- | --- | --- |
| 0 | 24.09±5.91 | (9.40,38.78) | 70.04 | <0.01^*^ | 3 |
| 5 | 47.17±7.00 | (29.78,64.56) |  |  | 3 |
| 10 | 70.00±5.59 | (56.13,83.88) |  |  | 3 |
| 20 | 89.72±4.78 | (77.85,101.59) |  |  | 3 |

*P* < 0.05^#^，*P* < 0.01^*^

Statistical anylasis on LDH level

| Concentration | ‾*x±S* | *95%CI* | *χ^2^* | *P* | N |
| --- | --- | --- | --- | --- | --- |
| 0 | 91.14±9.53 | (67.48,114.80) | 13.23 | 0.010^*^ | 3 |
| 1 | 128.16±19.10 | (80.71,175.61) |  |  | 3 |
| 5 | 207.24±20.58 | (156.13,258.36) |  |  | 3 |
| 10 | 259.79±51.79 | (131.13,388.45) |  |  | 3 |
| 20 | 371.36±23.71 | (312.46,430.27) |  |  | 3 |

^△^Heterogeneity of variance, rank sum test with Kruskal-Wallis was performed for analysis among groups. *P* < 0.05^*^，*P* < 0.01^#^

Figure S18. Confidence interval has been reported on n=3 sample size.
